# Synergistic and individual actions of nanoemulsified vegetable oil and betaine as natural growth promoters on broiler performance, serum proteins, lipid profile, and safety enzymes

**DOI:** 10.1371/journal.pone.0333285

**Published:** 2025-10-09

**Authors:** Maged A. Al-Garadi, Gamaleldin M. Suliman, Elsayed O. Hussein, Abdullah N. Al-Owaimer, Ayman A. Swelum, Nabil A. Almalamh, Rashed A. Alhotan, Mohammed M. Qaid

**Affiliations:** Department of Animal Production, College of Food and Agriculture Sciences, King Saud University, Riyadh, Saudi Arabia; Universitas Sebelas Maret, INDONESIA

## Abstract

This study evaluates the effects of betaine (Betafin^®^; BET) and soybean oil nano-emulsion (Magic Oil^®^, NEPO) and their combination (NEPO+BET) as natural drinking water supplements on broiler chickens during the finisher phase. A trial was conducted using 320 Ross 308 broilers divided into four groups: control (no NEPO, no BET), NEPO only (1 mL/L water), BET only (1 g/L water), and their combination (both together; NEPO+BET). Growth performance parameters were assessed at 21, 28, and 35 days, with serum biochemical analyses performed at day 35. Results indicated that NEPO and BET individually improved growth rate and feed efficiency without increasing feed intake and improved metabolic indices, such as reducing low-density lipoprotein cholesterol, risk of hypercholesterolemia ratio, creatinine, and alkaline phosphatase compared with control, while the combined treatment (NEPO+BET) yielded the most pronounced effects. Thus, NEPO+BET treatment significantly gave the best FCR, albumin, globulin, triglycerides, and very-low-density lipoprotein values (*p* < 0.05) compared to other groups, suggesting synergism in enhancing feed efficiency, enhancing protein metabolism and lipid homeostasis. BET independently lowered serum glucose. Liver and kidney function markers remained within normal ranges, indicating the safety of these additives. These findings support the use of NEPO+BET as effective growth promoters in poultry nutrition through improved nutrient utilization. Future studies should explore the molecular mechanisms involved and the long-term implications of their use in commercial poultry production.

## Introduction

The finisher phase of broiler production is characterized by rapid muscle growth and increased metabolic demands, necessitating diets rich in energy and protein to support optimal performance [1–4]. However, such formulations can induce metabolic stress by increase oxidative load, nitrogen turnover, and hepatic stress, leading to oxidative imbalance, gut health challenges, and compromised immune function in broilers [5]. This type of metabolic stress may occur by stimulating excessive mitochondrial activity and reactive oxygen species (ROS) production, thereby increasing oxidative load and taxing antioxidant defenses [6,7]. Simultaneously, elevated protein intake accelerates nitrogen turnover, requiring heightened deamination, ammonia synthesis, and urinary excretion—all of which impose additional demands on the liver and kidneys, contributing to hepatic stress [6,8]. With increasing consumer demand for antibiotic-free poultry, the industry is exploring natural feed additives that enhance growth, immunity, and overall health while reducing reliance on synthetic growth promoters [9,10].

Among the promising alternatives, betaine – a methyl donor known for its osmoregulatory and metabolic benefits [11] – and plant-derived essential oils with antimicrobial and antioxidant properties have gained considerable attention [12,13]. These additives not only improve feed efficiency and stress resilience but also contribute to sustainable poultry production by enhancing nutrient utilization and reducing nitrogen excretion [11–14]. The nanoemulsified soybean oil (NEPO) and betaine (BET) are two promising candidates for improving broiler performance under diverse production conditions. Essential oils (EOs) and their nanoemulsified derivatives, such as soybean-derived compounds, have demonstrated antimicrobial, antiparasitic, and antioxidant properties while enhancing gut health, digestive enzyme activity, and feed efficiency [15–18].

Soybean oil, although not a phytogenic compound, was nanoemulsified to enhance its bioavailability and functional properties, including solubility, dispersion stability, and intestinal absorption, thereby enhancing its functional value as an energy-dense and bioactive feed additive [19,20]. Unlike phytogenic essential oils valued for antimicrobial and antioxidant effects, NEPO primarily improves lipid digestibility and nutrient utilization, with added metabolic benefits. This study clarifies that NEPO was used for its enhanced bioavailability and efficiency rather than as a phytogenic compound, with the inclusion level based on prior evidence of safe and effective use in poultry diets [9,21]. Nanoemulsification technology further enhances the bioavailability and efficacy of essential oils, making them more effective in poultry production [9,22]. Meanwhile, betaine, a naturally occurring trimethyl derivative of glycine found in sugar beets and other plants, functions as both an osmolyte and a methyl donor [23–25]. It has been shown to regulate water balance, improve protein metabolism, reduce lipid deposition, and enhance carcass quality in broilers [1,10,26,27].

Previous research suggests that betaine’s methyl donor properties may complement the anti-oxidative [28,29] and lipid-modulating effects of NEPO [30], potentially leading to synergistic improvements in broiler performance. Thus, combining NEPO and BET is essential due to their complementary roles in improving lipid profiles and protein metabolism-key factors in optimizing metabolic efficiency, energy utilization, and overall growth performance. This method was selected because water intake is more consistent than feed intake, ensuring uniform delivery, easier dosage adjustment, and rapid administration without the need for diet reformulation. Thus, water-based supplementation is simple to apply and offers greater flexibility, precision, and potential bioavailability of NEPO and BET [14,31,32]. The rationale for combining NEPO with BET is supported by our recent study [33], which demonstrated their beneficial effects on carcass traits and meat quality characteristics of broilers under stress conditions. While previous studies only evaluated NEPO or BET separately in feed [34–37], little or none in drinking water supplements [38], under optimal housing conditions, particularly regarding key health parameters such as lipid profiles and protein metabolism.

Various water- and feed-based supplements have been applied in modern poultry production as alternatives to antibiotics to enhance growth performance and health [39]. Moreover, supplementation of bioactive compounds via drinking water is a practical approach in commercial poultry production, particularly under osmotic pressure or environmental stress conditions when water intake increases substantially. Water-based delivery ensures rapid absorption, circumvents potential reductions in feed intake, and allows more uniform distribution of additives across the flock [39,40]. In this situation, the inclusion of BET in drinking water, although less common than dietary inclusion, is justified by its physicochemical stability in aqueous systems and its proven biological activity as a methyl donor and serve as osmolytes to conserve water and maintain cell integrity regulate osmotic pressure [41,42]. Similarly, NEPO can be efficiently administered via water due to its enhanced solubility and stability in colloidal form, ensuring better bioavailability compared with conventional oil supplementation [43–45]. Thus, delivering NEPO and BET through drinking water may represent a practical and efficient strategy to improve nutrient utilization in broilers.

This study aims to bridge this knowledge gap by evaluating the individual and combined effects of soybean oil nano-emulsion and betaine as natural drinking water supplements on growth performance, serum biochemistry, and health indicators in broiler chickens during 21–35 d. The findings will provide valuable insights into the potential of these natural supplements to enhance broiler production while supporting the industry’s shift toward sustainable, antibiotic-free systems.

## Materials and methods

### Ethical statement

This study was carried out in strict accordance with the Committee on the Ethics of Animal Experiments, King Saud University (Protocol Number: KSU-SE-21–02). No anesthesia or analgesia was required as no surgical procedures were performed. Daily welfare checks and predefined humane endpoints were applied to minimize distress. All procedures, including weighing and volume and frequency of blood sampling, were conducted in line with ethical best practices to reduce stress and pain, following the 3Rs principle (Replacement, Reduction, Refinement), in compliance with Institutional Animal Care and Use Committee (IACUC) regulations. Birds were sacrificed by cervical dislocation in accordance with halal principles and the KSU Ethics Committee approval.

### Study design and management

A total of 320 straight-run day-old Ross 308 healthy broiler chicks with similar average body weights (46.35 ± 0.06 g) to ensure uniformity in growth potential were used in this experiment. Birds obtained from obtained from AL-Khumasia company and immunized at hatchery. The birds were managed under uniform housing conditions at animal production department, KSU (24°43′28.8″N 46°37′07.9″E), and fed a standard commercial diet along 35 days. The experimental diets were formulated to meet the nutrient requirements of broilers according to NRC (1994) [46] guidelines and Aviagen recommendations for Ross 308 during the starter (0–10 days), grower (11–20 days), and finisher (21–35 days) phases (Table 1).

**Table 1:** Composition of experimental diets and nutrients analysis as a fed %.

Ingredients	Starter (0–10 days)	Grower (11–20 days)	Finisher (21–35 days)
**Corn grain**	57.65	61.32	60.64
**Soybean Meal48% Crude protein**	35.44	31.79	30.48
**Palm oil**	2.06	2.61	4.63
**Dicalcium phosphate**	1.92	1.73	1.42
**Limestone**	1.06	0.96	1.42
**Common salt**	0.36	0.36	0.35
**L-Threonine**	0.3	0.12	0.07
**Methionine**	0.38	0.33	0.28
**L-Lysine HCl**	0.28	0.22	0.15
**Choline Cl70**	0.06	0.06	0.06
**Minivet premix 0.5%** ^**^	0.5	0.5	0.5
**Nutrients**	**Analysis**		
**Dry matter %age**	89.61	89.58	85.66
**ME kcal/kg**	3000	3070	3200
**CP %**	22.4	20.7	19.5
**Arginine %**	1.52	1.399	1.273
**Lysine %**	1.435	1.29	1.131
**Methionine %**	0.711	0.641	0.566
**Methionine + Cysteine %**	1.08	0.99	0.882
**Threonine %**	1.143	0.9	0.803
**Tryptophan %**	0.277	0.254	0.228
**Valine %**	1.017	0.951	0.901
**Ether extract**	4.601	5.256	7.236
**Linoleic acid %**	1.597	1.714	4.496
**Crude fiber%**	2.65	2.589	2.523
**Calcium %**	0.96	0.87	0.937
**Total phosphorus %**	0.713	0.663	0.625
**Available phosphorus %**	0.48	0.44	0.40
**Sodium %**	0.16	0.16	0.16

On day 21, chicks were weighed and a completely randomized design (CRD) was assigned to four experimental groups with similar average body weights (1029 ± 0.89 g). Supplementation was initiated at 21 days of age, coinciding with the onset of the finisher phase when lipid deposition and protein turnover are most active. Although many feed additives require prolonged exposure to elicit metabolic effects, previous findings [37] demonstrated that betaine supplementation significantly improved growth performance and efficiency during 21–42 days of age, while its impact during the starter and grower phases was minimal. This evidence supports the rationale for commencing supplementation at day 21. Each group comprised eight replicates, with ten birds per replicate. The experimental groups were a 2 × 2 factorial design resulting in four groups: A0B0, A1B0, A0B1, A1B1 (0 = absent, 1 = present). Factor A: NEPO and Factor B: BET. Thus, the experimental groups were as follows:

1. **Control Group (no NEPO, no BET; A0B0)**: No drinking water supplements.
2. **NEPO Group** (**A1B0)**: Drinking water supplemented with Nanoemulsified plant oil (NEPO only) at 1 mL/L.
3. **BET Group (A0B1)**: Drinking water supplemented with Betaine (BET only) at 1 g/L.
4. **NEPO+BET Group (both together; A1B1)**: Drinking water supplemented with a combination of NEPO (1 mL/L) and BET (1 g/L).

The NEPO (Magic oil^®^, ATCO Pharma, Egypt) was supplemented in drinking water at a level equivalent to 1 mL of the commercial product per liter of water, corresponding to 0.985 mL (985 mg/mL) nano-emulsified crude soybean oil (98.5%) together with polysorbate-80, and vitamin as described in Suliman et al. [14]. Betaine was provided as Betafin^®^ (Danisco Animal Nutrition, UK, part of IFF – International Flavors & Fragrances), which contains 91% anhydrous betaine. To achieve an effective dosage of 1 g of betaine per liter of water as described in Khajali; and Faraji [47], the required supplementation was 1.1 g of BET/L. Both supplements were easily soluble in water, ensuring uniform delivery across replicates. The dosage of 1 g/L betaine was determined according to manufacturer specifications (Betafin®, 91% purity) and supported by previous broiler trials demonstrating growth and metabolic benefits [33–35,37,38]. NEPO was provided at 1 mL/L, a level chosen based on preliminary stability tests and earlier reports on nanoemulsified phytogenic oils in poultry nutrition [14,33,36,38]. These dosages were selected to reflect both practical application and scientific precedent.

Birds were reared in floor cages under controlled environmental conditions, with a temperature starting at 33°C on day 0 and gradually reduced to 24°C by day 20. A lighting program of 23 hours on and 1 hour off was maintained throughout the experiment. All birds were provided *ad libitum* access to feed and water.

### Performance measurements

Body weight and feed intake were recorded for each replicate group at 21, 28, and 35 days. Average daily gain (ADG), daily feed intake (DFI), and feed conversion ratio (FCR) were calculated for the finishing period (21–35 days) and its sub-phases (21–28 days and 29–35 days).

### Serum biochemistry

At day 35, two birds from each replicate (*n* = 16 per group) were randomly selected for blood sampling. Blood was collected from the wing vein into sterile tubes, centrifuged at 3000 rpm for 10 minutes, and the serum was stored at −20°C until analysis. Serum biochemistry analyses were performed in the poultry health laboratory of the Department of Animal Production, King Saud University. Biochemical parameters, including total protein (TP), albumin, glucose, total lipids (TL), total cholesterol, triglycerides, high-density lipoprotein cholesterol (HDL), and low-density lipoprotein cholesterol (LDL), were measured using enzymatic colorimetric kits (RANDOX, UK) and a spectrophotometric analyzer (UDICHEM 310, KSA). Enzymatic markers of liver function, including alkaline phosphatase (ALP), aspartate aminotransferase (AST), and alanine transaminase (ALT), as well as kidney function markers (urea and creatinine), were also assessed.

Serum globulin = serum TP levels – serum albumin levels [48]. Albumin: globulin ratio = serum albumin/ serum globulin. According to Friedewald equation [49], low-density lipoprotein cholesterol (LDL) was calculated as following: LDL = (Total cholesterol) − (HDL) − (triglycerides/5). HDL/LDL ratio, risk of hypercholesterolemia ratio = LDL/total cholesterol, and very-low-density lipoprotein (vLDL = triglycerides/5) were estimated as reported by Friedewald et al. [50] and Attia et al. [51]. All the measurements were taken in duplicate.

### Statistical analysis

The data were analyzed with the General Linear Model (GLM) procedure in SAS software (Version 9.3, SAS (52) Institute): The model used for analysis was a 2 × 2 factorial design two-way ANOVA [52].


$${\text{Y}}_{\text{ijk}}=\;\mu +\;{\text{A}}_{\text{i}}+\;{\text{B}}_{\text{j}}+{\left(\text{A}\times \text{B}\right)}_{\text{ij}}\;+\;\epsilon_{\text{ijk}}$$

Where Y_ijk_ represents the observed variable, μ is the overall mean. Factor A_i_ is the NEPO effects (i; absent *vs* present), factor B_j_ is the BET effects (_j;_ absent *vs* present), resulting in 2 × 2 factorial arrangements, and ε_ijk_ is the random error. The design follows with: Interaction test (A × B “NEPO×BET”) first: If interaction significant, it was interpreting simple effects (NEPO effect at each level of BET, and *vice versa;* the combination is additive or interactive). The interaction is the difference of differences: Interaction = (ῩA1B1- ῩA0B1) – (ῩA1B0 - ῩA0B0). If interaction not significant, it was interpreting main effects of NEPO and BET. If this is ≠ 0 (statistically), A’s effect changes when B is present *vs* absent. Experimental unit **n* *= 8 cage of animals for performance indices and **n* *= 16 serum blood samples per group (two birds from each replicate) for biochemistry analysis. Randomization was used to allocate experimental units (cages) to control and treatment groups. A computer-generated randomization sequence was created using [Microsoft Excel], and stratified randomization based on initial body weight was applied to ensure uniform distribution across groups and to minimize selection bias. Potential confounders such as treatment order, cage location, and measurement timing were controlled using a randomized cage allocation strategy, standardized environmental and management conditions, and a balanced sampling order. All measurements were taken by the same trained veterinarian personnel at consistent time points to minimize bias. Assumptions of normality and homogeneity of variances were checked using the Shapiro-Wilk and Levene’s tests, respectively.

A priori power analysis was conducted considering expected variability, effect size, and desired statistical power (higher than 80%). All inclusion and exclusion criteria were established a priori based on standard practices in poultry research. All of the birds were in good health; thus, no bird along the trial was excluded. Extreme values (outliers) that significantly deviated from the group mean were excluded. Treatment means were compared using Tukey’s post hoc test, and statistical significance was set at *p* < 0.05. Pearson correlation analysis of performance and biochemical parameters was performed to clarify and evaluate relationships among measured indices, improve interpretation of treatment effects, and provide a more integrated understanding of growth and metabolic responses. All values were expressed as statistical means ± standard error.

## Results

### Growth performance

As shown in Table 2, the dietary supplementation with NEPO and/or BET significantly (*p* < 0.05) enhanced the growth performance of broiler chickens compared to the control. During 21–28 days, the NEPO and NEPO+BET groups had the highest ADG (approximately 110 g/bird/d), significantly (**p* *< 0.05) higher than the control (94 g/bird/d), while BET showed an intermediate value (102 g/bird/d). During 28–35 days, the NEPO+BET and BET groups had the highest ADG (108.8 and 104.3 g/bird/d, respectively), significantly (*p* < 0.05) higher than the control (91.5 g/bird/d), while NEPO had an intermediate value (99.7 g/bird/d). During the overall period (21–35 days), both supplements, either individually or in combination, significantly (**p* *< 0.05) increased ADG (109.2, 104.8, and 103.4 g/bird/d for NEPO+BET, NEPO alone, and BET alone, respectively) compared with the control (92.8 g/bird/d). Feed intake was not significantly (**p* *> 0.05) different among treatments during the finisher period.

**Table 2:** Effect of betaine (BET), nanoemulsified plant oil (NEPO), and their combination (NEPO+BET) in drinking water on performance indicators of broilers.

Parameters	Treatments				Standard error	Probability
	Control	NEPO	BET	NEPO+BET		
**Final body weight (g)**	2,312^c^	2,486^b^	2,498^ab^	2,561^a^	19.57	<0.0001
**Average daily gain (g/bird/d)**						
**Days 21–28**	94.13^c^	109.94^a^	102.51^b^	109.69^a^	1.489	<0.0001
**Days 28–35**	91.51^c^	99.73^ab^	104.26^a^	108.77^a^	3.25	0.006
**Days 21–35**	92.82^b^	104.83^a^	103.39^a^	109.23^a^	1.510	<0.0001
**Daily feed intake (g/bird/d)**						
**Days 21–28**	145.84	149.13	147.16	145.22	1.48	0.276
**Days 28–35**	166.05	165.74	166.65	162.39	1.44	0.178
**Days 21–35**	155.94	157.43	156.90	153.80	1.162	0.155
**Feed conversion ratio (g: g)**						
**Days 21–28**	1.56^a^	1.36^bc^	1.44^b^	1.32^c^	0.025	<0.0001
**Days 28–35**	1.82^a^	1.66^b^	1.60^b^	1.49^c^	0.067	0.006
**Days 21–35**	1.69^a^	1.50^b^	1.52^b^	1.41^c^	0.024	<.0001

a–c Means within a row with a common superscript differ significantly (*p <* 0.05). *n* = 8 replicated cage/treatment.

Across the periods of 21–28, 28–35, and 21–35 days, both supplements, whether administered individually or in combination, significantly (**p* *< 0.05) improved FCR, with the combined treatment exerting the strongest (synergistic) effect compared with the individual supplement or control. Between 21 and 28 days, the NEPO+BET group achieved the best (**p* *< 0.05) FCR (1.32), followed by NEPO (1.36) and BET (1.44), whereas the control group had the poorest value (1.56). From 28–35 days, NEPO+BET (1.49) outperformed BET (1.60) and NEPO (1.66), while the control again showed the worst FCR (1.82). Over the entire period (21–35 days), the NEPO+BET group maintained superior (**p* *< 0.05) efficiency (1.41), compared with NEPO (1.50) and BET (1.52), with the control remaining the least efficient (1.69).

**Fig 1:**
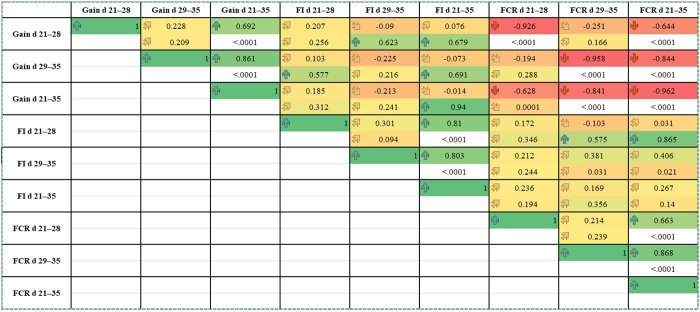
Pearson correlation coefficients between performance indices of broilers treated with BET, NEPO, and their combination (NEPO +BET) in drinking water (*n* = 32; Prob > |r| under H0: Rho = 0).

The correlation coefficients between the performance indices of broiler chickens supplemented with BET, NEPO, and NEPO+BET are presented in Fig 1. A highly significant (**p* *< 0.05) negative correlation was observed between average body weight gain and FCR at 21−28 days (R^2^ = −0.926), 29−35 days (R^2^ = −0.958), and 21–35 days (R^2^ = −0.962), indicating that greater weight gain was consistently associated with improved feed efficiency under prolonged supplementation. In contrast, there are no observed significant correlation between feed consumption (*p* > 0.05) with ADG or with FCR.

### Serum biochemical parameters

#### Serum proteins and glucose levels.

The results for serum proteins and glucose concentrations are presented in Table 3. Regarding serum proteins parameters, NEPO+BET supplementation significantly increased globulin levels (1.57 vs. 1.18 g/dL) leading to significantly increased total protein levels (3.30 *vs*. 2.87 g/dL) and reduced albumin-to-globulin ratio (1.09 *vs*. 1.47 g/dL) compared to the control group. While individual NEPO and BET supplementation resulted in moderate serum protein levels. Glucose levels remained within normal ranges and were lower (*p* < 0.05) in the NEPO group (123.8 mg/dL) compared to the other groups.

**Table 3:** Effect of betaine (BET), nanoemulsified plant oil (NEPO), and their combination (NEPO+BET) in drinking water on serum proteins and glucose levels at 35 days in broilers.

Parameters	Treatments				Standard Error	Probability
	Control	NEPO	Betaine	NEPO+BET		
**Total protein (g/dl)**	2.87^b^	2.98^ab^	3.10^ab^	3.30^a^	0.123	0.049
**Albumin (g/dl)**	1.69	1.64	1.59	1.71	0.079	0.6925
**Globulin (g/dl)**	1.18^c^	1.34^bc^	1.51^ab^	1.59^a^	0.075	0.0013
**A: G Ratio**	1.47^a^	1.29^ab^	1.10^b^	1.09^b^	0.079	0.0031
**Glucose (mg/dl)**	133.2^a^	129.8^ab^	123.8^b^	135.4^a^	2.713	0.0220

a–c Means within a row with a common superscript differ significantly (p < 0.05). A: G Ratio Albumin: Globulin Ratio. n = 16 sample/treatment.

#### Lipids profile.

The results for serum lipid profile are presented in Table 4. Birds receiving NEPO and/or BET, particularly, NEPO+BET, had significantly (**p* *< 0.05) lower LDL, and total lipid concentrations and improved HDL/LDL ratio. In addition, birds receiving NEPO+BET had significantly (**p* *< 0.05) lower vLDL (15.84 vs. 17.64 mg/dL) and triglycerides (79.19 vs. 88.12 mg/dL) concentrations, reducing the risk of hyperlipidemia ratio in broilers (by 29.06%) compared to the control and even to an individual treated groups.

**Table 4:** Effect of betaine and/or nanoemulsified plant oil (NEPO), and their combination (NEPO+BET) in drinking water on lipids profile at 35 d in broilers.

Parameters (mg/dl)	Treatments				Standard error	Probability
	Control	NEPO	BET	NEPO+BET		
**Total lipids**	433^a^♣^^	397^b^	414^ab^	393^b^	9.09	0.011
**Triglycerides**	88.21^a^	87.04^a^	85.32^a^	79.19^b^	1.879	0.006
**Cholesterol**	130.0	113.7	116.9	114.1	5.29	0.110
**HDL**	57.76	58.86	64.50	64.80	3.71	0.404
**LDL**	54.57^a^	37.46^b^	35.29^b^	33.50^b^	3.42	0.0001
**vLDL**	17.64^a^	17.41^a^	17.06^a^	15.84^b^	0.376	0.006
**HDL/ LDL ratio**	1.13^b^	1.93^a^	1.98^a^	1.97^a^	0.171	0.001
**RHCHOL**	0.413^a^	0.322^b^	0.304^b^	0.293^b^	0.019	0.0002

Abbreviations: HDL: High density lipoprotein cholesterol; LDL: Low density lipoprotein cholesterol “LDL = Total Cholesterol − HDL) − (triglycerides/5)”, RHCHOL: Risk of hypercholesterolemia ratio = LDL/total cholesterol, and vLDL (triglycerides/5). ^♣^*n* = 16 samples. ^a–c^Means within a row lacking a common superscript differ significantly (*p* < 0.05).

#### Safety indicators.

The results for serum kidney and liver function markers on day 35 are presented in Table 5. Urea levels remained stable across all groups. Serum creatinine was significantly (**p* *< 0.05) lower in the BET (0.404 mg/dl) and BET+NEVO (0.474 mg/dl) compared with control (0.646 mg/dl), while NEVO alone was an intermediate value (0.615) (P = 0.0036). Relative to the control, BET and NEPO+BET reduced creatinine levels. Furthermore, liver function markers (Liver enzymes: ALT and AST) were unaffected by treatments (*p* > 0.05) and remained within normal physiological ranges across all treatment groups. While alkaline phosphatase (ALP) activity was highest in the control group (*p* = 0.0344). ALP levels were reduced in the treated groups particularly, NEPO+BET group.

**Table 5:** Effect of betaine (BET) and/or nanoemulsified plant oil (NEPO), and their combination (NEPO+BET) in drinking water on liver and kidney functions at 35 d in broilers.

Parameters	Treatments				Standard Error	Probability
	Control	NEPO	BET	NEPO+BET		
**Kidney function**						
Urea (mg/dl)	6.88	6.42	6.29	6.77	0.215	0.1801
Creatinine (mg/dl)	0.646^a^	0.615^ab^	0.404^b^	0.474^b^	0.051	0.0036
**Liver enzymes**						
ALT (U/L)	216.80	211.82	209.43	207.68	3.055	0.1806
AST (U/L)	20.67	20.90	20.71	20.62	0.182	0.314
ALP (U/L)	487.4^a^	445.4^b^	451.1^ab^	431.7^b^	13.54	0.0344
ALT:AST Ratio	10.02	10.14	10.12	10.09	0.174	0.9674

Abbreviations: ALT: alanine transaminase; AST: aspartate aminotransferase; ALP: alkaline phosphatase. *n* = 16 birds. ^a,b^ Means within a row lacking a common superscript differ significantly (*p* < *0.05*).

**Fig 2:**
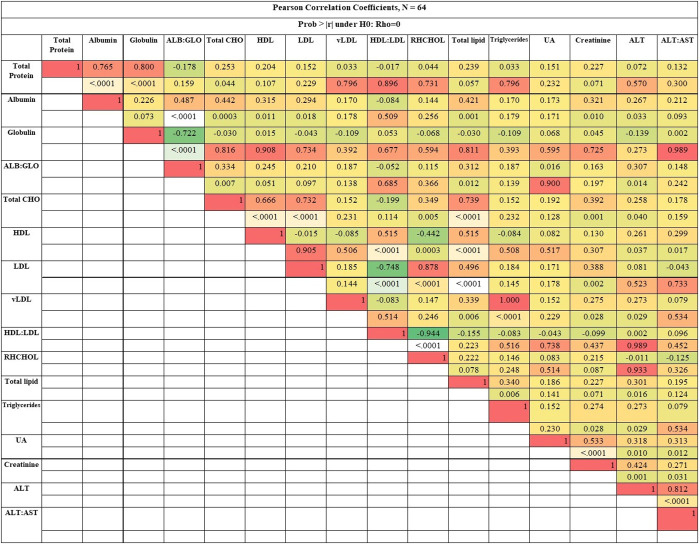
Pearson correlation coefficients between different blood biochemical parameters of broilers treated with betaine (BET) and/or nanoemulsified plant oil (NEPO), and their combination (NEPO+BET) in drinking water. Abbreviations: CHO: cholesterol; HDL: high density lipoprotein cholesterol; LDL: low density lipoprotein cholesterol, RHCHOL: Risk of hypercholesterolemia ratio; ALT: alanine transaminase; AST: aspartate aminotransferase. (Other parameters not provided here showed no meaningful correlation.).

The results for correlation coefficients analysis between blood biochemical parameters at day 35 of broiler chickens treated with betaine and/ or Nano emulsified plant oil are presented in Fig 2. There are significant positive correlations were observed between total protein and levels of globulin (R^2 ^= 0.80) and albumen (R^2 ^= 0.77). Serum albumin/globulin ratio significantly (*p* < 0.0001) correlated negatively (R² = −0.72) with serum globulin level.

Total lipids correlated positively (R² = 0.74; *p* < 0.0001) with total cholesterol level, particularly HDL concentrations (R² = 0.52; *p* < 0.0001). Triglyceride levels correlated strongly positively (R² = 1.00; *p* < 0.0001) with very low-density lipoprotein cholesterol (vLDL) levels. HDL/LDL ratio significantly (*p* < 0.0001) correlated negatively (R^2 ^= −0.75) with LDL level and positively (R^2 ^= −0.52) with LDL level. Hypercholesterolemia ratio correlated positively (R² = 0.878) with LDL level.

Serum urea levels correlated weakly positively (R² = 0.53; *p* < 0.0001) with serum creatinine levels. Serum ALT/AST ratio significantly (*p* < 0.0001) correlated positively (R² = 0.72) with serum ALT level. Furthermore, ALP levels showed a negative correlation with total protein.

## Discussion

This study evaluated the synergistic and individual effects of BET and NEPO as natural drinking water supplements on the growth performance and serum biochemistry of broilers. BET or NEPO can be added to the drinking water of chickens [38,53]. However, their combined effects under optimal housing conditions had not been extensively studied. These findings suggest that NEPO and BET can serve as promising natural feed additives to improve broiler health and productivity while maintaining metabolic balance and organ function.

The present study confirms that delivering NEPO and BET through drinking water is a feasible and biologically relevant approach. During environmental and osmotic stresses, water intake increases markedly, often exceeding feed intake, thereby ensuring that bioactive compounds administered in water are consistently ingested and effectively absorbed [54,55]. This route also bypasses potential reductions in DFI, a common consequence of thermal stress, which can otherwise limit the efficacy of feed-based additives. Importantly, betaine is stable in aqueous solutions and retains its functional role as a methyl donor and osmolyte during osmotic stress and dehydration when delivered via water [41,42], supporting its effectiveness in improving protein metabolism and mitigating oxidative stress. Likewise, NEPO maintain dispersion stability in water and improve lipid digestibility and absorption [38,43–45]. Collectively, these findings and supporting evidence justify the use of drinking water as a practical vehicle for NEPO and BET supplementation in commercial poultry systems, particularly for improving resilience and performance.

The improved feed efficiency and weight gain observed in the NEPO+BET group highlight the potential of NEPO in enhancing nutrient absorption and metabolism. According to studies by Klasing et al. [56], both villus length and duodenal osmolality increased when BET was added at concentrations of 0.05% and 0.1%. In addition, Kettunen et al. [57] found that the addition of 0.2% BET to the diet helped the duodenum maintain water balance. These findings align with previous reports demonstrating that phytogenic feed additives and osmolytes in poultry nutrition enhancing broiler performance and metabolic health [58,59]. The observed improvements in feed efficiency and growth performance are consistent with studies by others [38,60,61], who reported that NEPO and probiotics or BET contribute to better nutrient absorption, metabolic function, and meat quality traits in the case of high temperatures inside broiler houses. However, some studies, such as Abudabos et al. [38], attributed the no significant improvement in ADG to differences in environmental conditions, diet composition, or genetic factors may influence the efficacy of these additives. Essential Oils (EOs) are hydrophobic natural materials containing secondary metabolites (i.e., monoterpenes, phenylpropeness and esquiterpenes). They are mainly produced by higher plants, especially aromatic ones (over 17,000 species) [62]. Campolo et al. [62] found that using EOs and surfactants (Tween 80) to create sonicated EO-based nano-emulsions (Sonicated Artemisia) had a significant impact on the nano-emulsion characteristics, which were the most stable over time, had the smallest droplets, and the best stability.

Biochemical parameters in serum can quickly reflect the physiological health status of the animal as well as metabolic and nutritional processes [63,64]. Significant changes in serum protein and lipid profiles highlight the metabolic benefits of NEPO+BET supplementation. NEPO+BET group showed increased globulin levels by 34.75% leading to increased total protein levels by 14.98%, consistent with their role in enhancing protein metabolism and immune responses [65]. The improvements in total protein and globulin levels indicate enhanced immune function, supporting the role of these additives in strengthening poultry health, aligning with studies that report natural additives’ role in stress mitigation, immune modulation, and enhance protein anabolism [66]. Although globulin levels increased, direct immune indicators such as antibody titers and cytokines were not assessed to validate this elevation. Future studies should therefore confirm the immunomodulatory effects using these specific biomarkers. In the NEPO group, serum glucose concentration decreased by 7.06%, showing that NEPO can contribute to hypoglycemia in blood. That decline may be due to sodium-glucose transporter 1 limiting glucose uptake on the absorptive surface of the intestine [67,68]. This work presented state-of-the-art nanosystems for phytochemicals in the treatment of *diabetes mellitus-* type 2, indicating the predominance and potential of nanotechnologies [69].

NEPO+BET group reduced total lipids by 41.62% and triglyceride by 10.23% through reducing undesirable cholesterol (LDL by 38.61%, vLDL by 10.20%,) suggests a positive influence on lipid metabolism, improving HDL/ LDL ratio by 74.34% and reducing the risk of hyperlipidemia in broilers by 29.06% compared to the control. In constant with Ghanima et al. [65], an increase in NEPO concentration decreased serum total lipid levels. The lipid-lowering effects of NEPO and BET, particularly their ability to reduce total lipids and LDL concentrations, align with previous studies demonstrating the hypolipidemic properties of natural emulsifiers and methyl donors [70,71]. These findings suggest that NEPO and BET may reduce the risk of hyperlipidemia, contributing to overall metabolic health in broilers. The observed improvement in the HDL/LDL ratio further supports their potential to enhance cardiovascular health in poultry. This indicates that the groups receiving natural treatments, particularly NEPO+BET, highlights potential modulating lipid metabolism and promoting cardiovascular health in broilers through reducing the risk of hypercholesterolemic ratio, potentially improving meat quality. According to Ibrahim et al. [72], EOs of *Citrus aurantifolia* leaves ameliorated dyslipidemia by dramatically lowering serum triacylglycerol, total cholesterol, and LDL levels while raising HDL levels. In addition, NEPO+BET stimulates the reductase enzyme of the cholesterol biosynthetic (HMG-CoA) in the liver. Phytosterols can influence the activity of HMG-CoA reductase, acetyl-CoA carboxylase, and malic acid enzymes [73]. The lipid-lowering effects of NEPO+BET observed in this study are in agreement with Adil et al. [74], highlighting the potential of NEPO to regulate cholesterol metabolism. Feeding rosemary essential oil in nanoprotected form in a 200 mg/kg diet could be used as an antibiotic substitute to improve broiler’s lipid profile, intestinal health, and immune-antioxidant status [74]. He et al. [75] reported consistent results in respect decreased BET supplementation to triglyceride, free fatty acids, and LDL levels and inconsistent results in respect decreasing HDL, emphasizing the need for further research to determine the optimal dosage and formulation.

The reduction in serum creatinine levels observed in the NEPO and NEPO+BET groups by 54.64% and 26.63% compared with control, indicates improved kidney function, likely due to the antioxidant and anti-inflammatory properties of NEPO [65]. The elevated insignificantly urea levels in the NEPO+BET group could indicate increased protein turnover or metabolic inefficiencies when the two additives are combined. While this effect did not impact growth performance, it raises questions about the metabolic interactions between NEPO and BET that may influence nitrogen metabolism. The unchanged levels of liver enzymes (ALT and AST) across all groups suggest that neither NEPO nor BET exerted any hepatotoxic effects on broilers, supporting their safety as aqueous supplements. While ALP was highest in the control group. The reductions in ALP levels and the positive correlation between ALT/AST ratio and ALT levels suggest improved hepatic function, emphasizing the hepatoprotective effects of NEPO.

Antioxidant supplementation has been shown to reduce protein and lipid oxidative instability, which may be related to increased cellular antioxidant enzyme activity [76]. In addition, the methyl donor and osmolyte betaine have antioxidant properties [77]. Sun et al. [78] observed that addition of BET and/or vitamin C as an antioxidant can mitigate the adverse effects of stressors and improve the performance and antioxidant capacity of broilers.

Under commercial poultry production systems, where birds often experience environmental and nutritional stressors, these natural supplements could play a crucial role in promoting health and reducing reliance on synthetic additives or antibiotics. Therefore, the findings support the hypothesis that NEPO can influence lipid metabolism more effectively than their traditional counterparts. The findings of this study shed light on the possibility of natural water supplements to improve broiler health and metabolism under thermoneutral circumstances. While NEPO and BET showed individual benefits in lowering serum lipids and improving protein metabolism, their combination (NEPO+BET) produced the best results, implying that the observed improvements in blood protein levels and hepatic function support NEPO+BET’s potential as a functional feed additive with both performance-enhancing and health-promoting properties.

A limitation of this study is the absence of a conventional antibiotic growth promoter as a positive control. While our results demonstrate clear benefits of NEPO, BET, and their combination compared with an un-supplemented control, future studies should benchmark these effects against standard antibiotic or synthetic growth promoters. Additionally, while the dosages were selected based on manufacturer guidelines and previous literature, dose-response studies are needed to optimize and validate the supplementation levels. The findings of this study on NEPO and betaine supplementation in broilers are likely to generalize well to other poultry species, especially those used in commercial production, but the applicability to non-avian species or other types of animals requires more research. While there may be indirect relevance to human biology, particularly in terms of lipid metabolism and supplement bioavailability, caution should be exercised when translating the results directly to humans due to significant species-specific physiological differences. Further research would be required to validate the outcomes in other animal models and human trials

## Conclusions

In conclusion, the study’s findings showing that NEPO and BET, either individually or in combination, reduced serum LDL, Risk of hypercholesterolemia ratio, and creatinine, while improving HDL/LDL ratio, growth rate, and FCR without increasing feed intake. This study provides compelling evidence for the synergistic benefits of NEPO+BET in broiler nutrition. The novel combination significantly improves FCR and broiler health by reducing the risk of hyperlipidemia, increasing albumin and globulin levels, and enhancing lipid metabolic through reducing triglycerides and vLDL, while maintaining normal liver and kidney functions, indicating synergistic effects on protein metabolism and lipid homeostasis. However, the mechanistic basis of synergy of NEPO+BET combination requires further investigation, and long-term studies considering nanoemulsion stability are warranted. Also, further dose-response trials and comparisons with synthetic growth promoters are needed to confirm the robustness of NEPO+BET supplementation. Overall, the research is a valuable contribution to the field of poultry science and has strong potential for practical implementation.
